# Draft genome sequencing data of a feather mite, *Amerodectes protonotaria* Hernandes 2018 (Acariformes: Proctophyllodidae)

**DOI:** 10.1016/j.dib.2022.108835

**Published:** 2022-12-16

**Authors:** Alix E. Matthews, Than J. Boves, Katie L. Percy, Asela J. Wijeratne

**Affiliations:** a College of Sciences and Mathematics and Molecular Biosciences Program, Arkansas State University, Jonesboro, Arkansas, United States; b Department of Biological Sciences, Arkansas State University, Jonesboro, Arkansas, United States; c Audubon Delta, National Audubon Society, Baton Rouge, Louisiana, United States; d United States Department of Agriculture, Natural Resources Conservation Service, Addis, Louisiana, United States

**Keywords:** Acari, Analgoidea, Coevolution, Illumina, Symbioses, Whole genome sequencing

## Abstract

Feather mites are ubiquitous, permanent, obligate ectosymbionts of avian hosts and are a valuable natural system for studying host-symbiont evolutionary and ecological dynamics at multiple levels of biological organization. However, a lack of a sequenced genome impedes molecular studies using this system. Therefore, we present the first draft genome of a symbiotic feather mite, *Amerodectes protonotaria* Hernandes 2018. The genome sequence data presented here were derived from an individual female mite that was collected in the field from *Protonotaria citrea*, its only known host species. Short read sequence data were obtained using an Illumina NovaSeq 6000 platform. From these data, we assembled a 59,665,063 bp draft genome consisting of 2,399 contigs. Raw short reads and the assembled genome sequence are available at the National Center for Biotechnology Information (NCBI)’s Sequence Read Archive (SRA) under BioProject PRJNA884722. The data presented here are beneficial for future research on the biology and evolution of closely related mites and the genomics of host-symbiont interactions.

**Specifications Table**

**Table:** 

Subject	Biodiversity
Specific subject area	Genomics, Symbioses
Type of data	Table Figure Raw DNA sequencing reads Genome assembly
How the data were acquired	Illumina NovaSeq 6000 150 bp paired-end reads Quality control: FastQC version 0.11.5 Trimming sequences: Cutadapt version 3.0 and BBMap version 38.82 Deduplicating sequences: BBMap version 38.82 Assembly: SPAdes version 3.12.0 Assembly statistics: QUAST version 5.0.0 Assembly completeness: BUSCO version 5.2.2
Data format	Raw Analyzed Assembled
Description of data collection	The feather sample was obtained from a wild-caught *Protonotaria citrea* host. A single mite was isolated from the feather and genomic DNA was extracted from the individual mite using Qiagen's QIAamp DNA Micro Kit.
Data source location	BREC's Bluebonnet Swamp Nature Center Baton Rouge, Louisiana United States of America 30.368102, -91.107749
Data accessibility	All data in this article are available through the National Center for Biotechnology Information (NCBI) BioProject PRJNA884722 (https://www.ncbi.nlm.nih.gov/bioproject/PRJNA884722). Raw sequence data are available under Sequence Read Archive (SRA) accession SRR21738601. This Whole Genome Shotgun project has been deposited at DDBJ/ENA/GenBank under the accession JAOZJS000000000. The version described in this paper is version JAOZJS010000000.

## Value of the Data

• We used Illumina paired-end raw reads to construct a draft genome for the feather mite, *Amerodectes protonotaria* Hernandes 2018 (Acariformes: Proctophyllodidae: Pterodectinae), a species used for research on symbiont evolution, ecology, and natural history.
• This dataset will be useful to evolutionary ecologists interested in coevolution and cophylogenetics of symbionts. It will also facilitate comparative genomic research and genome-wide analyses of Analgoidea and other related species.
• This draft genome can be used as a reference genome for genomic and evolutionary studies of mites, particularly avian mites (including skin, nasal, and nest mites). For example, these data can be used to test hypotheses related to the genomics and evolution of host specificity and permanent symbiotic lifestyles.
• This is the first draft genome for an analgoid outside of the family Pyroglyphidae. Although pyroglyphids (e.g., house dust mites) are medically important and descendants of feather mites [1], many are biologically differentiated from all other members of Analgoidea (e.g., free-living versus permanent ectosymbionts). Thus, our *A. protonotaria* draft genome is the first of non-model, permanent mites within this superfamily.

## 1 Objectives

Vane-dwelling feather mites (Acari: Acariformes: Astigmata: Analgoidea, Pterolichoidea) are ubiquitous avian ectosymbionts and represent a useful natural system to study symbiont natural history, coevolution, and host-symbiont ecological dynamics at the species, population, and individual levels. However, until now, no sequenced genome existed for feather mites. This has impeded the potential for molecular, genomic, and evolutionary studies using this system. To address this gap in knowledge, our objective was to assemble a draft genome for the feather mite, *Amerodectes protonotaria* Hernandes 2018.

## 2 Data Description

**Fig. 1:**
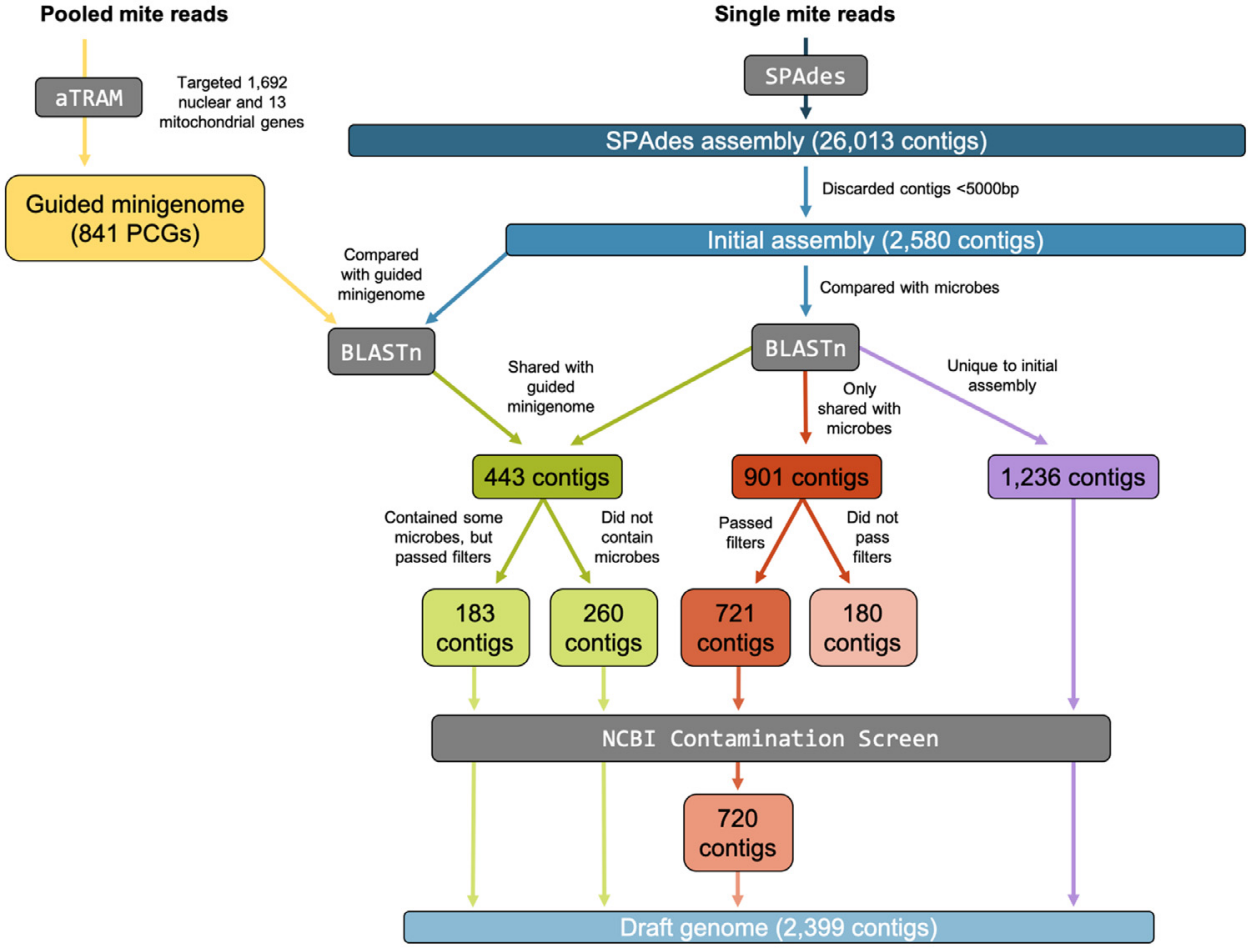
Flowchart of quality assessment and control of the draft genome. Bioinformatic programs/steps are in gray boxes with white text, each assembly leading to the final draft genome is in sequentially shaded blue boxes with white text, and the number of contigs (or protein coding genes [PCGs] in the case of the guided minigenome) included at each step is in colored boxes with black text. Text boxes adjacent to arrows explain considerations at each step.

The data reported here describe the draft genome sequencing and assembly of a single individual female feather mite of the species *Amerodectes protonotaria* Hernandes 2018 (Acariformes: Proctophyllodidae: Pterodectinae)*.* Our initial sequencing data consisted of 8,037,920 short reads; after read quality processing, 5,048,079 reads remained for assembly. The draft genome was assembled into contigs using SPAdes version 3.12.0 [2] and went through several levels of quality assessment and control measures (Fig. 1). After these steps, we retained a 59.6Mb (59,665,063 bp) genome consisting of 2,399 contigs with an N50 of 43,948 bp and GC content of 39.99% (Table 1). The largest contig was 462,516 bp (Table 1). The total length of the draft genome is within the expected range of the three available reference pyroglyphid mite (Acariformes: Pyroglyphidae) genomes. Specifically, the American house dust mite (*Dermatophagoides farinae*) reference genome is 58.8 Mb [3], the European house dust mite (*Dermatophagoides pteronyssinus*) reference genome is 70.8 Mb [4,5], and the reference genome deposited for Mayne's house dust mite (*Euroglyphus maynei*) is 43.4 Mb [6]. The total length of the draft genome for *A. protonotaria* is also within range of the genomes deposited for *Sarcoptes scabiei* (Acariformes: Sarcoptidae), the mite that causes the disease scabies (56.2 Mb [7]; 56.6 Mb [8] 57.3 Mb [9]), as well as a number of other acariform mites [10].

**Table 1:** Genomic statistics of the *Amerodectes protonotaria* draft genome.

SRA	SRR21738601
BioProject	PRJNA884722
BioSample	SAMN31063905
GenBank Accession	JAOZJS000000000
BUSCO completeness (Arachnida)	93.4% complete
(Total BUSCOs used as references from the arachnida_odb10 database: 2,934)	91.7% single-copy; 1.7% duplicated, 1.8% fragmented, 4.8% missing
BUSCO completeness (Arthropoda)	87.9% complete
(Total BUSCOs used as references from the arthropoda_odb10 database: 1,013)	86.7% single-copy; 1.2% duplicated, 3.9% fragmented, 8.2% missing
Total number of contigs	2,399
Number of contigs (>= 5000 bp)	2,399
Number of contigs (>= 10000 bp)	1,491
Number of contigs (>= 25000 bp)	613
Number of contigs (>= 50000 bp)	276
Largest contig	462,516 bp
Total length	59,665,063 bp
Total length (>= 0 bp)	59,665,063 bp
Total length (>= 1000 bp)	59,665,063 bp
Total length (>= 5000 bp)	59,661,590 bp
Total length (>= 10000 bp)	53,163,946 bp
Total length (>= 25000 bp)	39,292,836 bp
Total length (>= 50000 bp)	27,465,648 bp
N50	43,948 bp
N75	18,291 bp
L50	327 bp
L75	869 bp
GC (%)	39.99
Number of N's per 100 kbp	2.98

To assess the completeness of the draft genome, the complete Benchmarking Universal Single-Copy Orthologs (BUSCO) was used and BUSCO score (C) was 93.4% for Arachnida (91.7% single-copy; 1.7% duplicated, 1.8% fragmented, 4.8% missing) and 87.9% for Arthropoda (86.7% single-copy; 1.2% duplicated, 3.9% fragmented, 8.2% missing; Table 1). The BLASTn of the draft genome to the Trouessartidae mitochondrial genome [11] resulted in 2 hits on a single contig with >75% identity covering 8,908 bp of the reference (e-values were both 0.0 and bit scores were 4065 and 706). These results suggest that our draft genome is fairly complete. This feather mite genome sequence will be a useful resource for future genomic, evolutionary, and ecological analyses of Analgoidea (both permanent and free-living) and other related mite species.

## 3 Experimental Design, Materials and Methods

### 3.1 Sample preparation and sequencing

*Amerodectes protonotaria* feather mites were collected from Prothonotary Warblers (*Protonotaria citrea* Boddaert 1783) in Louisiana, USA in June 2021. Mites were later isolated from feathers under a stereomicroscope and preserved in 95% ethyl alcohol at -20°C until DNA extraction. Genomic DNA (gDNA) was isolated from a single female *Amerodectes protonotaria* mite using the QIAamp DNA Micro Kit (Qiagen).

Short-read whole genome library preparation and sequencing were performed at the Roy J. Carver Biotechnology Center, University of Illinois at Urbana-Champaign. The gDNA library was constructed from up to 10ng of DNA after sonication with a Covaris ME220 (Covaris, MA) to an average fragment size of 400 bp using the UtraLow Input DNA Library Construction kit (Tecan, CA). The library was amplified with 8 PCR cycles and run on a Fragment Analyzer (Agilent, CA), and was quantified by qPCR on a BioRad CFX Connect Real-Time System (Bio-Rad Laboratories, Inc. CA). Sequencing was performed on an Illumina NovaSeq 6000 SP lane with 2 × 150 bp paired-end reads. The fastq read files were generated with the bcl2fastq v2.20 Conversion Software (Illumina, San Diego, CA).

### 3.2 *De novo* genome assembly and assessment

The short reads were trimmed of Illumina sequencing adapters using Cutadapt version 3.0 [12] with a Phred score quality threshold of 30. We further trimmed the 3’ end of the reads to 140 bases and deduplicated them using BBDuk (https://sourceforge.net/projects/bbmap/). The final reads were then analyzed using FastQC version 0.11.5 (Babraham Bioinformatics).

The reads were *de novo* assembled using SPAdes using the ‘–only-assembler’ flag and k-mer sizes of 21, 33, 55, 77, 99, and 111 (“SPAdes assembly” herein). Short contigs (<5000 bp) were discarded (number of contigs discarded = 23,433) from the SPAdes assembly and the resulting assembly (“initial assembly” herein) underwent further quality assessment.

For quality assessment and control, we first assembled orthologous genes (“guided minigenome” herein) of a pooled *A. protonotaria* DNA sample (unpublished BioProject PRJNA882441) using the automated Target Restricted Assembly Method (aTRAM) version 2.3.4 [13]. Specifically, we identified orthologous 1:1 single copy protein coding genes (PCGs; Acari: *Sarcoptes scabiei*), consisting of 1,692 target nuclear PCGs, using OrthoDB version 10.1 [14]. To target the 13 PCGs of the mitochondria, we used data from *Proctophyllodes miliariae*, which was the most closely related species to *A. protonotaria* with all 13 mitochondrial genes present in a reference dataset [15]. With amino acid translated nuclear and mitochondrial genes as targets, we ran aTRAM using SPAdes for five iterations. We ran an additional post-processing script within aTRAM that uses Exonerate version 2.2.0 [16] to identify and stitch together exon regions for each gene assembled. Genes that were ≥95% complete were selected for the final guided minigenome (n = 841 genes). We made a local Basic Local Alignment Search Tool (BLAST; [17]) database of our initial assembly and performed BLASTn searches against the guided minigenome.

Second, we identified and removed potential contaminants in the initial assembly using the guided minigenome as a template to retain contigs. To do so, we first downloaded bacterial (April 2022), fungal (May 2022), and archaeal (May 2022) genomes from the National Center for Biotechnology Information (NCBI) Reference Sequence (RefSeq) database. We then made local BLAST databases of each microbe and performed BLASTn searches against the initial assembly. Manual assessment and strict filtering of the contigs followed the first two BLASTn searches. Contigs that had hits neither to the guided minigenome nor any microbes (i.e., were unique to the assembly alone) were retained for the final reference (n = 1,236). Contigs that were shared between the guided minigenome and initial assembly with ≥80% query coverage and had high percent identity (>95%, or >92% if query coverage was >99%) were compared to the microbe databases (n = 443). Of these 443 contigs, those that contained no microbe matches were retained for the final reference (n = 260). The remaining 183 contigs matched with one or more microbes and were manually assessed. We retained contigs with low microbe query coverage (<1%). We more closely inspected the 5 (fungi) and the 9 (bacteria) contigs with ≥1% query coverage (zero contigs had ≥1% query coverage to Archaea). We followed strict filtering criteria to retain those that had relatively low percent identity (<80%) and/or short query alignment lengths that began at the same position (e.g., indicative of horizontal gene transfer). After these assessments, all 183 contigs were retained. Lastly, we assessed contigs that were shared only between the microbes and initial assembly (i.e., were not present in the guided minigenome; n = 901). We used more conservative query coverage cutoff values that were set by the maximum query coverage for each microbe identified in the guided minigenome-assembly BLAST filtering steps described above (bacteria: ≤7%, fungi: ≤4%, archaea: ≤0.8%). Those that passed the query coverage filters were retained if they had very short alignment lengths, despite the percent identity value. A total of 721/901 contigs passed these filters. After this, the genome underwent NCBI's Contamination Screen and all contigs but one were retained. Thus, a total of 2,399 contigs were kept for our final assembly (“draft genome” herein).

To ensure we captured the mitochondrial genome in the draft genome, we performed a BLASTn of the draft genome against a feather mite (Trouessartidae: *Trouessartia rubecula*) mitochondrial genome (14,125 bp; [11]). We then assessed the draft genome statistics and contiguity metrics with Quality Assessment Tool for Genome Assemblies (QUAST) version 5.0.0 [18]. Lastly, we identified the presence of core eukaryotic protein coding genes with BUSCO version 5.2.2 (Arthropoda and Arachnida sets; [19]) to further evaluate our draft genome quality and completeness. All bioinformatics pipelines and scripts are available on GitHub (https://github.com/alixmatthews/ASU_Apro_genome_assembly).

## Ethics Statements

Birds were captured and handled under the United States Geological Survey Bird Banding Laboratory permit #23805 and under the BREC Scientific Research permit CONS-2017-04.

## CRediT Author Statement

**Alix E. Matthews:** Conceptualization, Formal Analysis, Writing – Original Draft; **Than J. Boves:** Conceptualization, Writing – Reviewing and Editing; **Katie L. Percy:** Investigation, Writing – Reviewing and Editing; **Asela J. Wijeratne:** Conceptualization, Writing – Reviewing and Editing.

## Declaration of Competing Interest

The authors declare that they have no known competing financial interests or personal relationships that could have appeared to influence the work reported in this paper.

## Data Availability

Amerodectes protonotaria Genome sequencing and assembly (Original data) (NCBI)

## References

[1] Klimov P.B., OConnor B. (2013). Is permanent parasitism reversible? - critical evidence from early evolution of house dust mites. Syst. Biol..

[2] Bankevich A., Nurk S., Antipov D., Gurevich A.A., Dvorkin M., Kulikov A.S., Lesin V.M., Nikolenko S.I., Pham S., Prjibelski A.D., Pyshkin A.V., Sirotkin A.V., Vyahhi N., Tesler G., Alekseyev M.A., Pevzner P.A. (2012). SPAdes: a new genome assembly algorithm and its applications to single-cell sequencing. J. Comput. Biol..

[3] Chen J., Cai Z., Fan D., Hu J., Hou Y., He Y., Zhang Z., Zhao Z., Gao P., Hu W., Sun J., Li J., Ji K. (2021). Chromosome-level assembly of *Dermatophagoides farinae* genome and transcriptome reveals two novel allergens Der f 37 and Der f 39. World Allergy Organ. J..

[4] Dermauw W., Van Leeuwen T., Vanholme B., Tirry L. (2009). The complete mitochondrial genome of the house dust mite *Dermatophagoides pteronyssinus* (Trouessart): a novel gene arrangement among arthropods. BMC Genomics.

[5] Waldron R., McGowan J., Gordon N., McCarthy C., Mitchell E.B., Doyle S., Fitzpatrick D.A. (2017). Draft genome sequence of *Dermatophagoides pteronyssinus*, the European house dust mite. Genome Announc.

[6] Rider S.D., Morgan M.S., Arlian L.G. (2017). Allergen homologs in the *Euroglyphus maynei* draft genome. PLoS One.

[7] S.D. Rider, M.S. Morgan, L.G. Arlian, Draft genome of the scabies mite, Parasites and Vectors. 8 (2015) 585. 10.1186/s13071-015-1198-2. PMC4641413 26555130

[8] Korhonen P.K., Gasser R.B., Ma G., Wang T., Stroehlein A.J., Young N.D., Ang C.S., Fernando D.D., Lu H.C., Taylor S., Reynolds S.L., Mofiz E., Najaraj S.H., Gowda H., Madugundu A., Renuse S., Holt D., Pandey A., Papenfuss A.T., Fischer K. (2020). High-quality nuclear genome for *Sarcoptes scabiei*—a critical resource for a neglected parasite. PLoS Negl. Trop. Dis..

[9] Xu J., Wang Q., Wang S., Huang W., Xie Y., Gu X., He R., Peng X., Wu S., Yang G. (2022). Comparative genomics of *Sarcoptes scabiei* provide new insights into adaptation to permanent parasitism and within-host species divergence. Transbound. Emerg. Dis..

[10] Gregory T.R., Young M.R. (2020). Small genomes in most mites (but not ticks). Int. J. Acarol..

[11] Esteban R., Doña J., Vierna J., Vizcaíno A., Serrano D., Jovani R. (2018). The complete mitochondrial genome of the feather mite *Trouessartia rubecula* Jablonska, 1968 (Astigmata: Analgoidea: Trouessartiidae). Mitochondrial DNA Part B Resour.

[12] Martin M. (2011). Cutadapt removes adapter sequences from high-throughput sequencing reads. EMBnet.J..

[13] Allen J.M., LaFrance R., Folk R.A., Johnson K.P., Guralnick R.P. (2018). aTRAM 2.0: an improved, flexible locus assembler for NGS data. Evol. Bioinforma..

[14] Kriventseva E.V, Kuznetsov D., Tegenfeldt F., Manni M., Dias R., Simão F.A., Zdobnov E.M. (2019). OrthoDB v10: sampling the diversity of animal, plant, fungal, protist, bacterial and viral genomes for evolutionary and functional annotations of orthologs. Nucleic Acids Res.

[15] Doña J., Sweet A.D., Johnson K.P., Serrano D., Mironov S., Jovani R. (2017). Cophylogenetic analyses reveal extensive host-shift speciation in a highly specialized and host-specific symbiont system. Mol. Phylogenet. Evol..

[16] Slater G.S.C., Birney E. (2005). Automated generation of heuristics for biological sequence comparison. BMC Bioinformatics.

[17] Altschul S.F., Madden T.L., Schäffer A.A., Zhang J., Zhang Z., Miller W., Lipman D.J. (1997). Gapped BLAST and PSI-BLAST: A new generation of protein database search programs. Nucleic Acids Res.

[18] Gurevich A., Saveliev V., Vyahhi N., Tesler G. (2013). QUAST: quality assessment tool for genome assemblies. Bioinformatics.

[19] Manni M., Berkeley M.R., Seppey M., Simão F.A., Zdobnov E.M. (2021). BUSCO Update: novel and streamlined workflows along with broader and deeper phylogenetic coverage for scoring of eukaryotic, prokaryotic, and viral genomes. Mol. Biol. Evol..

